# Supplementary material for: Magnesium in subaqueous speleothems as a potential palaeotemperature proxy
Source: Nat Commun. 2020 Oct 6;11:5027. doi: 10.1038/s41467-020-18083-7 (PMC7538886; doi:10.1038/s41467-020-18083-7)
Supplement: Supplementary file 1 — Supplementary Information [file 41467_2020_18083_MOESM1_ESM.pdf]

Supplementary Information for

Magnesium in subaqueous speleothems as a potential palaeotemperature proxy

Drysdale et al.

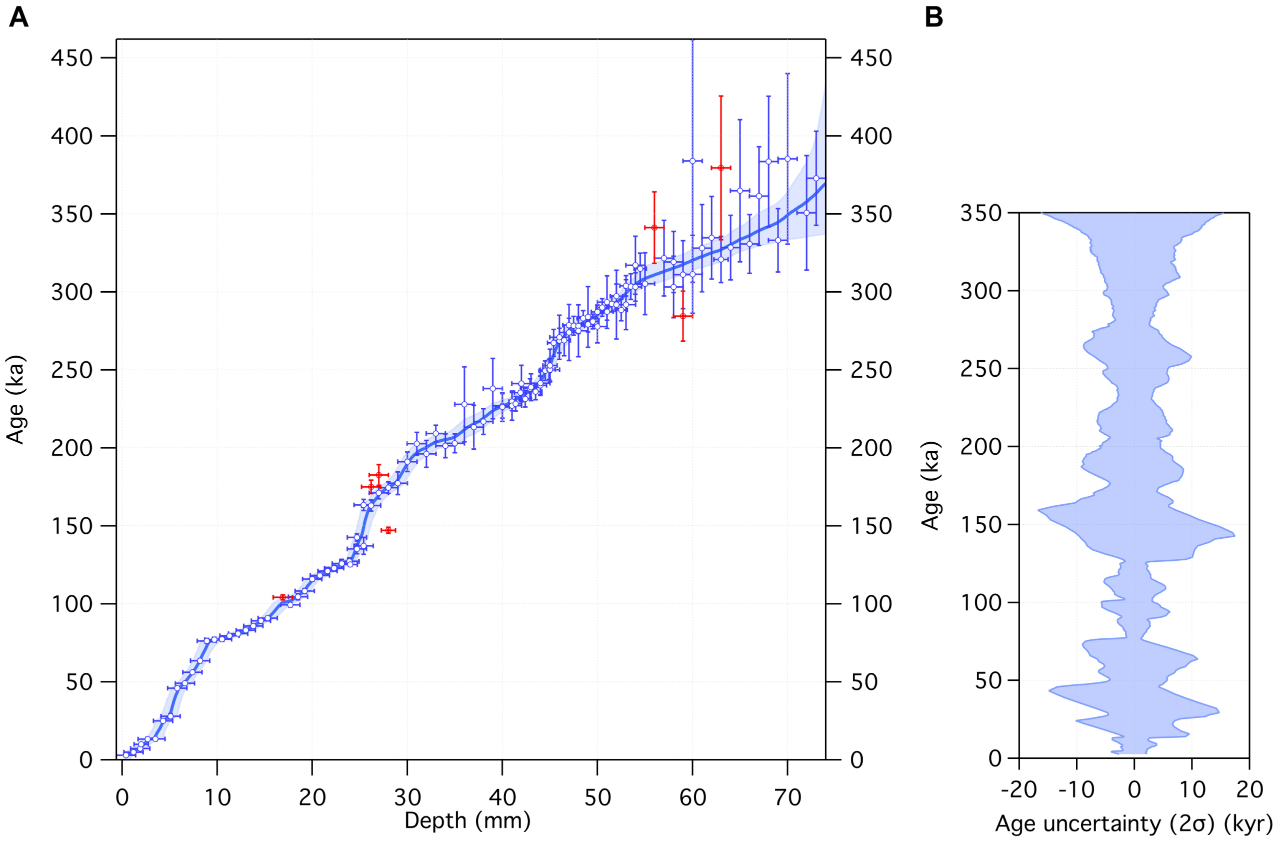

Supplementary Figure 1: Age-depth modelling of CD3-1. (A) Finite-positive-growth-rate model[1-2] for 114 U-Th ages from CD3-1. The vertical error bars and the blue-shaded envelope represent 2 s.d. uncertainties on the U-Th ages and the age model, respectively. The thick blue line is the age model used to interpolate the stable isotope and Mg data to the age scale. The red symbols represent seven U-Th ages considered to be outliers (see Supplementary Data 1). (B) A time series of age uncertainty variations derived from the age modelling.

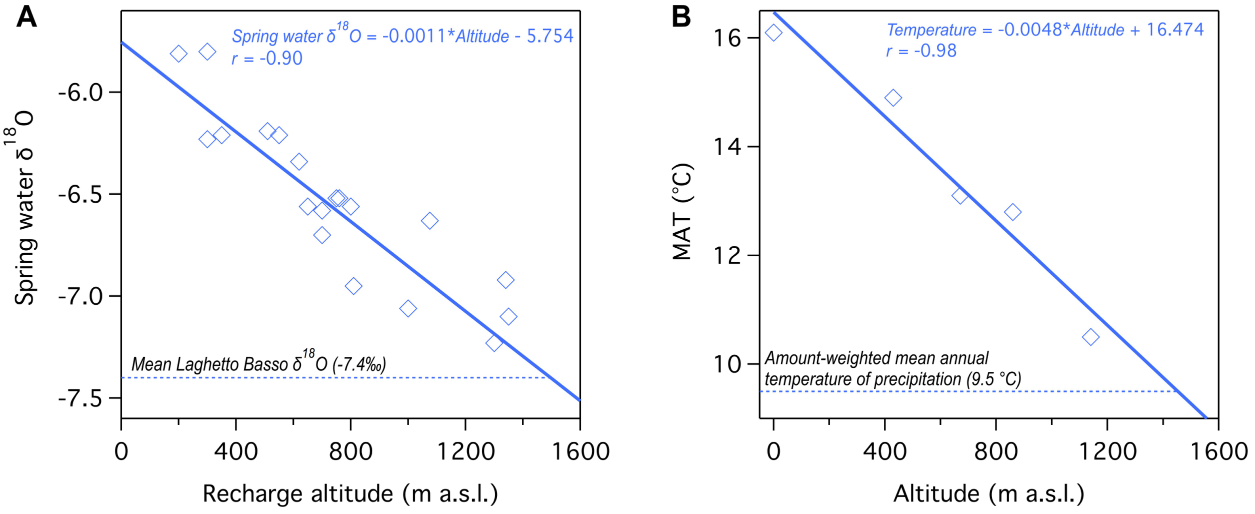

Supplementary Figure 2: Water isotope-altitude and temperature-altitude gradients. (A) Spring-water $\delta^{18}O$ values versus the mean altitude of spring recharge area for the Alpi Apuane. The altitude corresponding to the mean $\delta^{18}O$ value of Laghetto Basso[3] (dashed line) is 1500 m a.s.l. (B) Temperature versus altitude from five stations on an altitudinal transect (Forti dei Marmi sea level; Azzano 430 m; Vallelunga 670 m; Pian della Fioba 860 m; Cervaiole 1140 m). The altitude corresponding to the amount-weighted mean annual temperature of precipitation at Corchia is 1450 m a.s.l. Data are for each station covers the period 15 May 2014 to 11 May 2020 and are from https://www.sir.toscana.it/. Pearson *r* values and linear regression equations are shown.

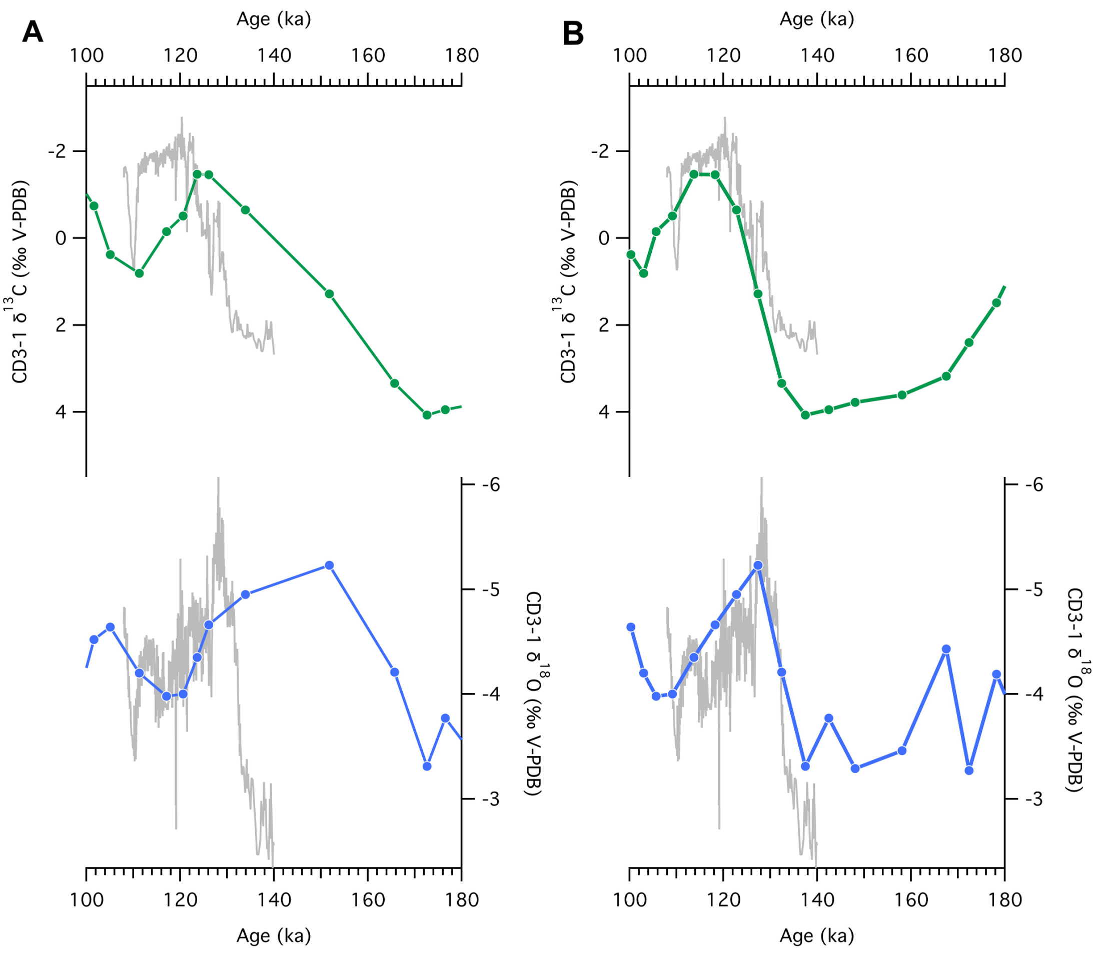

Supplementary Figure 3: **CD3-1 and stalagmite data for Marine Isotope Stage 6 – 5c.** (A) Low-resolution $\delta^{13}C$ (green) and $\delta^{18}O$ (blue) series and the corresponding $\delta^{13}C$ and $\delta^{18}O$ series from published stalagmite records from the same cave (grey) (re-interpolated to the most recent CCSS-18 age model[4]). Each time series is on its own age model. (B) Same as in (a) except that the age model for CD3-1 is based on synchronisation to the Iberian margin ocean-core chronology (see Methods).

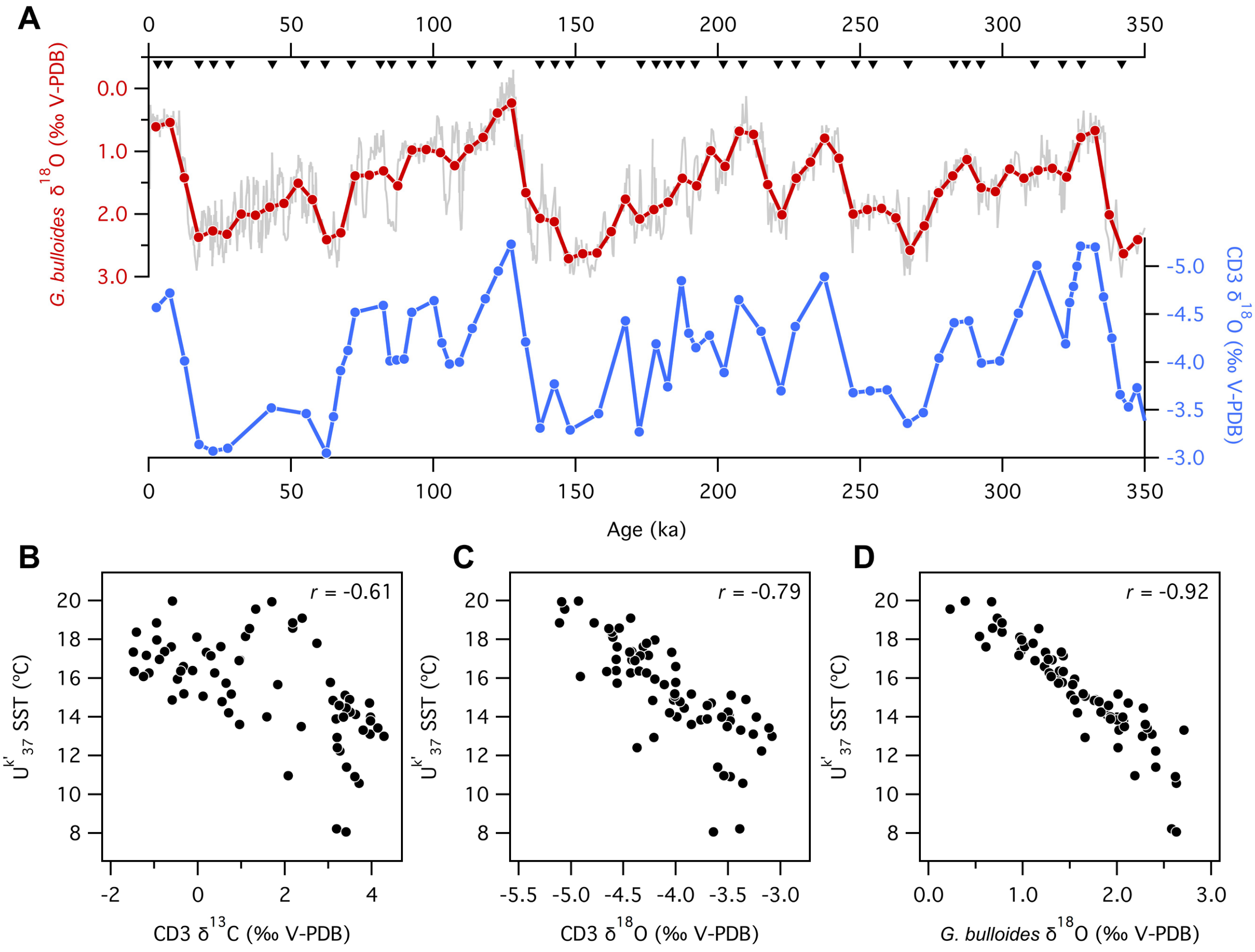

Supplementary Figure 4: Synchronisation of CD3-1 and ocean-sediment records. (A) Synchronisation of CD3-1 $\delta^{18}O$ to the planktic $\delta^{18}O$ from the Iberian margin[4-10]. The thin grey line is the original planktic $\delta^{18}O$ series whilst the heavy burgundy line is a 5-kyr resampling of the original. The blue line is the CD3-1 $\delta^{18}O$ which has been resampled at the same 5-kyr increment. The black triangles show the position of the tie points established in *Analyseries*[11] (see Methods). (B) – (D) Scatterplots of sea-surface temperatures versus CD3 $\delta^{13}C$, CD3 $\delta^{18}O$ and planktic (*G. bulloides*) $\delta^{18}O$ following the synchronisation shown in (A). All Pearson *r* values shown are statistically significant ($p < 0.05$; $df = 68$)

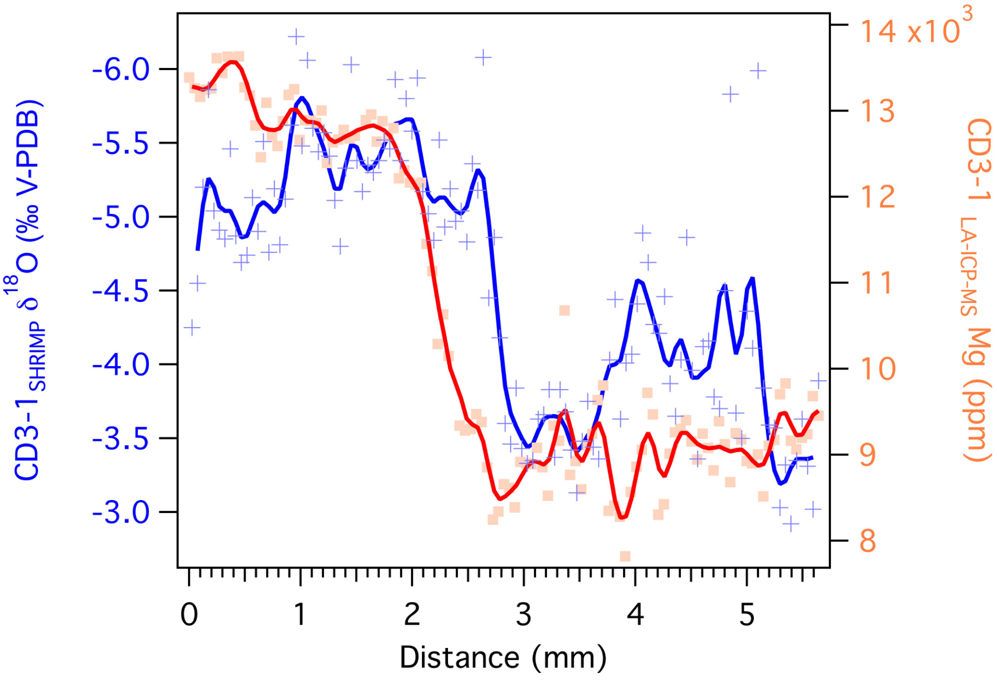

Supplementary Figure 5: High-resolution CD3-1 data through Termination II. Results from the SHRIMP-SI $\delta^{18}O$ (blue/light blue) and LA-ICP-MS Mg (red/pink) analyses on CD3-1 through Termination II. The markers are the raw data points and the solid lines are 3-point Gaussian smoothing splines.

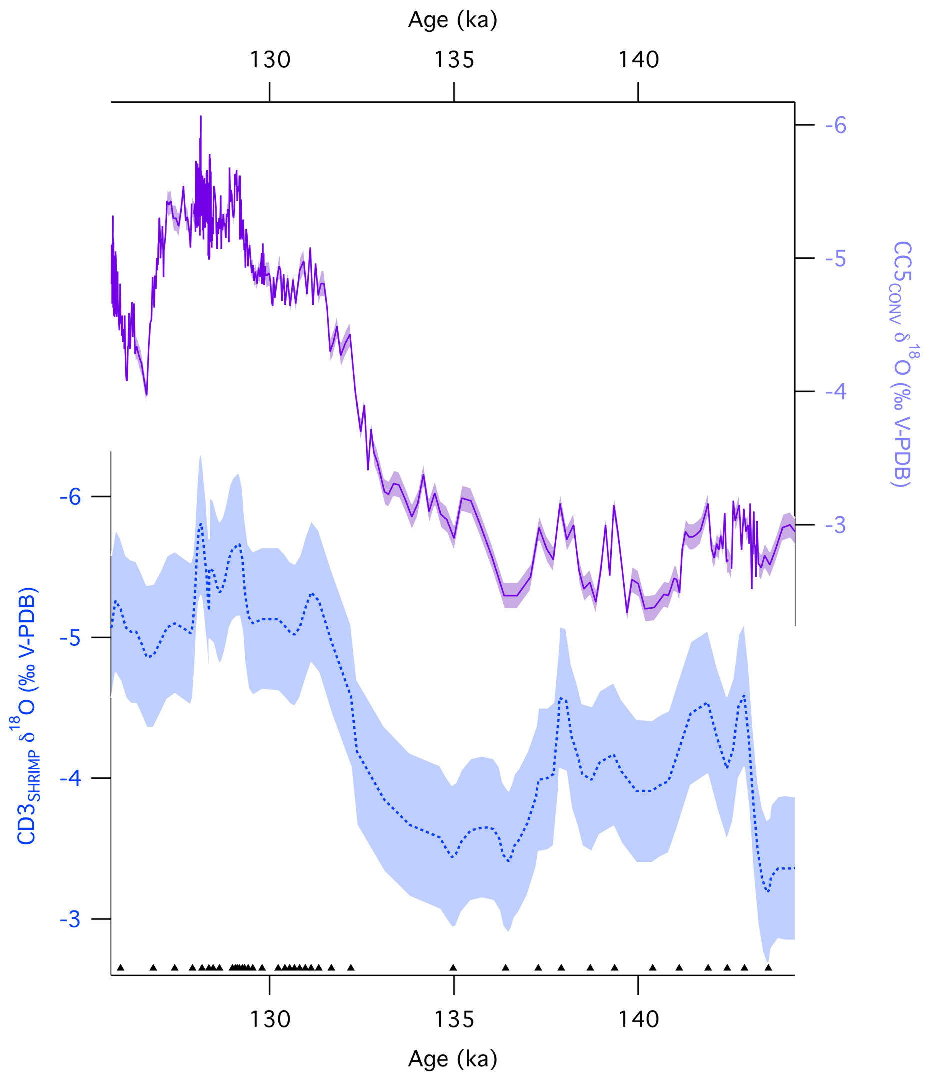

Supplementary Figure 6 : **Synchronisation of CD3-1 to the CCSS-18 stack.** The high-resolution SHRIMP-SI $\delta^{18}O$ CD3-1 is synchronised to the conventionally measured $\delta^{18}O$ from the Corchia Cave Speleothem Stack (CCSS-18)[4]. The mauve and light blue shaded envelopes are the 1 s.d analytical uncertainties for each series. The black triangles show the position of the tie points established in *Analyseries*[11] (see Methods for explanation).

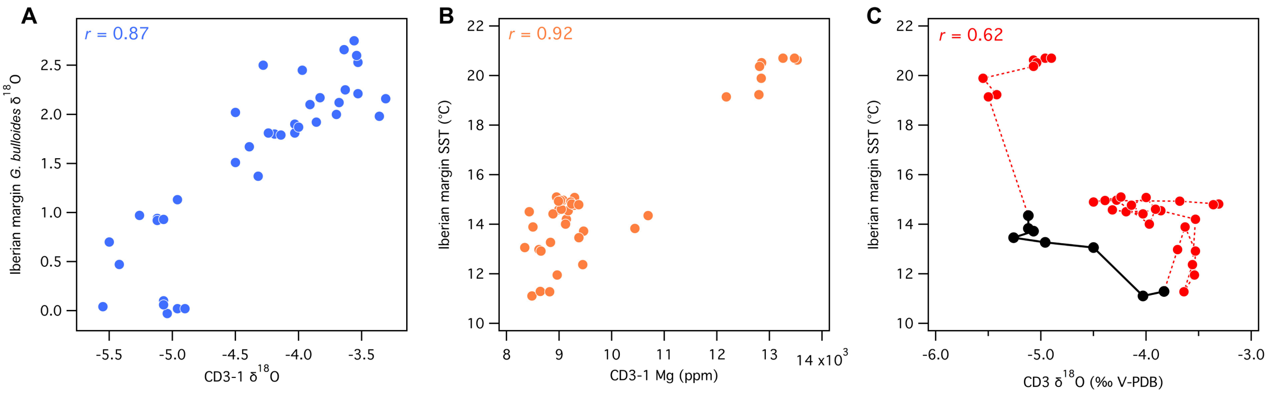

Supplementary Figure 7: Scatterplots of CD3-1 and ocean data through Termination II. (A) Planktic $\delta^{18}O$ (*G. bulloides*)[4,6-10] versus CD3-1 SHRIMP-SI $\delta^{18}O$. (B) Alkenone $U^{k'}_{37}$ sea-surface temperatures[4-10] versus CD3-1 Mg. (C) Alkenone $U^{k'}_{37}$ sea-surface temperatures[4-10] versus CD3-1 SHRIMP-SI $\delta^{18}O$. In the case of (C), the lines connecting the data points show the chronological sequence (bottom right – oldest; top left – youngest). The black lines and symbols are the data points corresponding to Heinrich Event 11. They show how the CD3-1 $\delta^{18}O$ is affected by the source effect due to deglacial meltwaters. This highlights that CD3-1 Mg (and not $\delta^{18}O$) is better at tracking sea-surface temperatures. Each series was resampled and binned at 500-yr increments (see Methods). All Pearson r correlation coefficients are statistically significant ($p < 0.05$; $df = 36$).

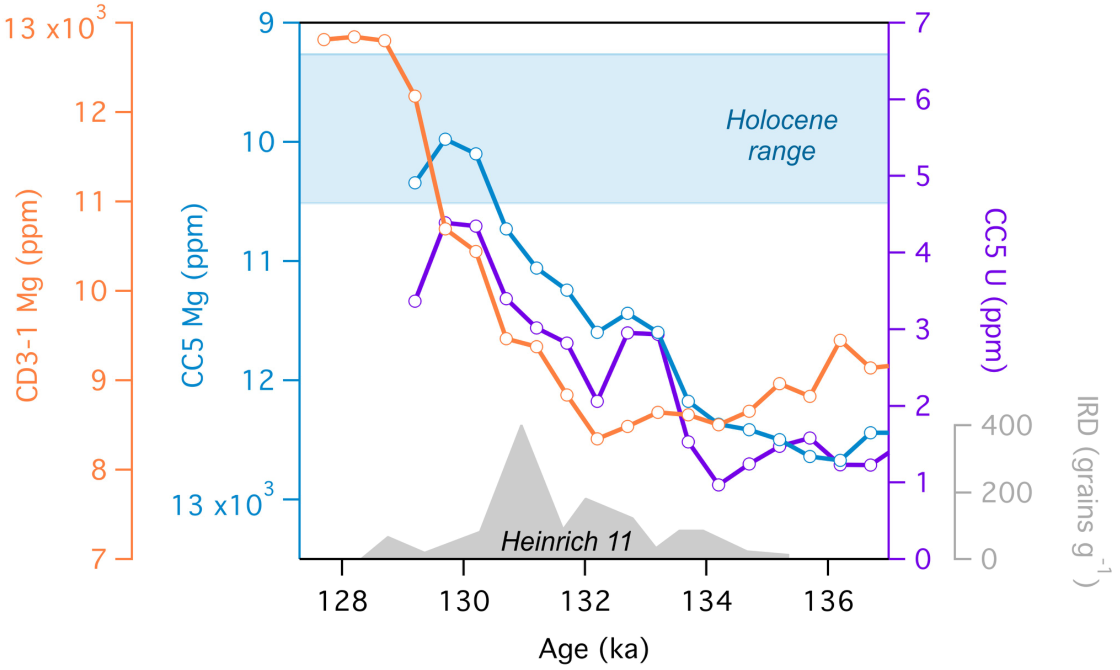

Supplementary Figure 8: CD3-1 and CC5 trace elements through Termination II. The CC5 Mg and U data[12] have been re-interpolated to the CCSS-18 age model[4]. (NB: CC5 is part of the CCSS-18 series reported in Tzedakis et al.[4] and is thus directly referenced to the CCSS-18 age model.) The Heinrich 11 ice-rafted debris series (IRD, grains per gram >90 µm) is from MD01-2444[13] and is plotted on the same age CCSS-18 age model[4]. All data has been resampled at 500-yr increments to reduce noise. The CC5 Mg *y*-axis has been inverted to highlight the hydrologically driven decrease through the termination. The raw CC5 data only extend to 128.5 ka[12], which excludes most of the Last Interglacial (LIG). Mg would be expected to decrease even further due to wet conditions over the first couple of thousand years of the LIG[4]. For comparison, the blue shaded band shows the Mg concentration range from stalagmite CC26[14] covering all but the last 120 years of the Holocene interglacial. To derive this range, the original series[14] was re-interpolated to a more recent age model[15] then resampled at 500-yr.

Supplementary References

1. Drysdale, R. N. *et al*. Palaeoclimatic implications of the growth history and stable isotope ($\delta^{18}O$ and $\delta^{13}C$) geochemistry of a Middle to Late Pleistocene stalagmite from central-western Italy. *Earth and Planetary Science Letters* **227**, 215–229 (2004).
2. Hendy, E. J., Tomiak, P. J., Collins, M. J., Hellstrom, J. C., Tudhope, A. W., Lough, J. M., & Penkman, K. E. H. Assessing amino acid racemization variability in coral intra-crystalline protein for geochronological applications. *Geochimica et Cosmochimica Acta* **86**, 338-353 (2012).
3. Daëron, M. *et al*. Most Earth-surface calcites precipitate out of isotopic equilibrium. *Nature Communications* **10**, 429 (2019).
4. Tzedakis, P. C. *et al*. Enhanced climate instability in the North Atlantic and southern Europe during the Last Interglacial. *Nature Communications* **9**, 4235 (2018).
5. Martrat, B., Grimalt, J. O., Shackleton, N. J., de Abreu, L., Hutterli, M. A. & Stocker, T. F. Four climate cycles of recurring deep and surface water destabilizations on the Iberian Margin. *Science* **317**, 502–507 (2007).
6. Tzedakis, P. C., Roucoux, K. H., de Abreu, L. & Shackleton, N. J. The duration of orest stages in southern Europe and interglacial climate variability. *Science* **306**, 2231–2235 (2004).
7. Vautravers, M. & Shackleton, N. J. Centennial scale surface hydrology off Portugal during Marine Isotope Stage 3: insights from planktonic foraminiferal fauna variability. *Paleoceanography* **21**, PA3004 (2006).
8. Tzedakis, P. C., Pälike, H., Roucoux, K. H. & de Abreu, L. Atmospheric methane, southern European vegetation and low-mid latitude links on orbital and millennial timescales. *Earth and Planetary Science Letters* **277**, 307–317 (2009).
9. Margari, V., Skinner, L.C., Tzedakis, P. C., Ganopolski, A., Vautravers, M. & Shackleton, N. J. The nature of millennial-scale climate variability during the past two glacial periods. *Nature Geoscience* **3**, 127–133 (2010).
10. Hodell, D., Crowhurst, S., Skinner, L., Tzedakis, P. C., Margari, V., Maclaghlan, S. & Rothwell, G. Response of Iberian Margin sediments to orbital and suborbital forcing over the past 420 kyr. *Paleoceanography* **28**, 1–15 (2013).
11. Paillard, D., Labeyrie, L. & Yiou, P. Macintosh program performs time-series analyses. *Eos Trans. AGU*, **77(39)**, doi.org/10.1029/96EO00259 (1996).
12. Drysdale, R. N. *et al*. Evidence for obliquity forcing of glacial Termination II. *Science* **32**5, 1527–1531 (2009).
13. Skinner, L. & Shackleton, N. Deconstructing Terminations I and II: revisiting the glacioeustatic paradigm based on deep-water temperature estimates. *Quaternary Science Reviews* **25**, 3312–3321 (2006).
14. Regattieri, E., Zanchetta, G., Drysdale, R., Isola, I., Hellstrom, J.C. & Dallai, L. Late glacial to Holocene trace element record (Ba, Mg, Sr) from Corchia Cave (Apuan Alps, central Italy): paleoenvironmental implications. *Journal of Quaternary Science* **29**, 381-392 (2014).
15. Bajo, P. *et al*. 'Cryptic' diagenesis and its implications for speleothem geochronologies. *Quaternary Science Reviews* **148**, 17-28 (2016).
16. Drysdale, R. N. *et al*. Precise microsampling of poorly laminated speleothems for U-series dating. *Quaternary Geochronology* **14**, 38–47 (2012).
17. Cheng, H. *et al*. Improvements in ^{230}Th dating, ^{230}Th and ^{234}U half-life values, and U-Th isotopic measurements by multi-collector inductively coupled plasma mass spectrometry. *Earth and Planetary Science Letters* **371–372**, 82–91 (2013).
18. Hellstrom, J. U-Th dating of speleothems with high initial 230Th using stratigraphical constraint. *Quaternary Geochronology* **1**, 289–295 (2006).
