# Supplementary material for: Magnesium in subaqueous speleothems as a potential palaeotemperature proxy
Source: Nat Commun. 2020 Oct 6;11:5027. doi: 10.1038/s41467-020-18083-7 (PMC7538886; doi:10.1038/s41467-020-18083-7)
Supplement: Supplementary file 3 — Description of Additional Supplementary Files [file 41467_2020_18083_MOESM3_ESM.pdf]

Description of Additional Supplementary Files

File Name: Supplementary Data 1
Description: Table of U-Th data for CD3-1 and Laghetto Basso pool water.

File Name: Supplementary Data 2
Description: Source data for Figures 2 to 5, and Supplementary Figures 1, 2, 4, 5 and 7
